# What is known about sexual and reproductive health in Latin American and Caribbean mining contexts? A systematic scoping review

**DOI:** 10.1186/s40985-017-0078-z

**Published:** 2018-01-05

**Authors:** Jose Wilches-Gutierrez, Patricia Documet

**Affiliations:** Department of Behavioral and Community Health Sciences, Graduate School of Public Health, University of Pittsburgh, 130 De Soto Street, Pittsburgh, PA 15261 USA

**Keywords:** Sexual health, Reproductive health, HIV, Mining industry, Latin America

## Abstract

**Background:**

Latin America and the Caribbean (LAC) has experienced an unprecedented mining boom since the mid-2000s with unknown effects on sexual and reproductive health (SRH). This study takes the essential first steps of summarizing the available literature regarding SRH in mining contexts in LAC, identifying critical gaps in knowledge, and discussing main implications for future research.

**Methods:**

We conducted a scoping review with a systematic search of health literature in four databases, reference lists of selected papers, and citations in Google Scholar.

**Results:**

The systematic search yielded 592 primary references and 16 articles from LAC. The 11 papers finally selected were conducted in gold-mining contexts in Brazil, Venezuela, Guyana, Peru, and Colombia, between 1995 and 2016. Ten studies centered on measuring HIV/STD prevalence among mineworkers and other populations; few examined associated risk factors. Eight studies reported high HIV/STD prevalence in the study population. None of the studies explored broader SRH issues.

**Conclusions:**

Available research is scarce and provides limited evidence on SRH in LAC mining contexts. Critical gaps include little knowledge on (1) broader SRH impacts besides HIV/STDs, (2) SRH in settings different from gold-mining contexts in Amazon countries, (3) mechanisms shaping SRH in LAC mining contexts, and (4) effective interventions in these scenarios. Future research must consider the distinctive demographic, environmental, socioeconomic, and gender dynamics triggered by the mining economy in the analysis of the relationship between mining and SRH, particularly in a period of extractive boom.

## Background

Consistent evidence characterizes mining as one of the world’s most hazardous activities because of the high risk of occupational accidents and toxicological exposure among mineworkers and communities [1]. Conversely, the current knowledge about the relationship between mining and sexual and reproductive health (SRH) is limited. The existing literature has been almost solely concerned with the spread of HIV, mostly in sub-Saharan Africa, where studies have observed that mining communities had a higher prevalence of HIV and other sexually transmitted diseases (STDs) than the general population [2, 3]. Some investigations found associations between having HIV/STDs and individual risk factors in mining contexts; these factors include single status, alcohol and drug use, history of previous STDs, partnership or sex with a mineworker, multiple sexual partners, paying for sex, transactional sex, and non-use of condoms [4–8]. While these studies focused mainly on groups considered at high-risk (typically mineworkers and sex workers), high prevalence of HIV/STDs and associations with similar risk factors have been described among other population groups in mining communities [4, 7, 9]. In general, this literature recognizes a relationship between the mining context and the dynamics of the HIV/STDs spread. Labor migration—linked to single-sex lodging and isolation and separation of miners from families—has been strongly associated with HIV transmission [2, 9–11]. Besides the spread of HIV/STDs, only a handful of studies have explored broader impacts of mining on SRH such as early marriage, increased fertility, short birth intervals, and lack of reproductive decision-making among local women [12–14].

### Mining boom in Latin America and the Caribbean

The study of the relationship between mining and SRH in Latin America and the Caribbean (LAC) is pertinent, as the region has experienced an unprecedented mining boom since the mid-2000s. The increased global demand for and rising prices of minerals [15], along with national mining laws favoring foreign investment [16], fueled the recent mining boom in the region. Between 2005 and 2010, the prices of coal, nickel, and copper doubled, and the price of gold increased five times between 2002 and 2011 [17]. In the same period, LAC received 24% of the global mining exploration budget, the largest investment for a single region [18]. As a result, mining production increased significantly, leading to a reactivation and intensification of operations in towns with established mining activities and, more significantly, the arrival of new mining endeavors in non-mining areas. Despite 50 to 70% of the regional mining production coming from industrial mining, most of the mining operations corresponded to artisanal and small-scale mining (ASM), which employed the majority of the region’s mining workforce [19]. In particular, illegal gold mining, predominantly of small scale, was estimated to employ directly around 350,000 people in Amazon countries and sustain about 500,000 people only in Bolivia and Peru [20].

Despite the high dividends from the mining sector commonly reported in the region (benefits for national economies remain debatable) [15, 21, 22], the mining boom has been associated with environmental, social, and health impacts at the local level, particularly affecting vulnerable populations. Studies and reports examining the impacts of mining in LAC have described environmental crises [20, 23, 24]; social conflicts and mobilization against extractive operations [25]; human rights violations to indigenous and Afro-descendant groups across the region [26]; child labor, sexual violence, sexual exploitation, and human trafficking linked to illegal gold mining in the Amazon [27, 28]; and environmental health effects, mental health impacts, and increased risks of infectious diseases, including STDs [29].

### Sexual and reproductive health in Latin America and the Caribbean

The mining boom in LAC takes place in a scenario of pending challenges in SRH. Estimations of either intimate partner violence or non-partner sexual violence against women vary between 24% in the Southern LAC and 41% in the Andean LAC—higher than the global estimation of 35% [30]. The region has the second highest rate of adolescent childbearing worldwide, 73 births per 1000 women aged 15–19 [31], and the highest estimated incidence of abortion and unsafe abortion, 44 and 31 abortions per 1000 women aged 15–44, respectively [32]. Most importantly, regional figures hide profound disparities in SRH between social groups. Lack of access to contraception, reproductive health services or maternal care, maternal mortality and morbidity, and sexual violence are higher among rural, low-income, low-educated, and ethnic minority women, principally indigenous and Afro-descendants, than the general population [31].

The recent mining boom in the region, in a scenario of profound SRH inequities, provides a strong rationale for examining to what extent the mining dynamics have impacted SRH at the local level. To this end, it is necessary to establish first what is currently known about the relationship between mining and SRH in the region. This scoping literature review takes the essential first steps of examining and summarizing the available literature regarding SRH in LAC mining contexts, identifying critical gaps in knowledge, and discussing primary implications for future research.

## Methods

A scoping review is a systematic process intended to explore the extent and nature of research on a particular topic, identify gaps in knowledge, and summarize and disseminate findings [33]. One of the key purposes of a scoping review is to provide an evidence background to guide future research, practice, and decision-making on the subject of interest [34]. A scoping review is particularly relevant when there is not a clear picture of the existing evidence regarding a field of research. That is, scoping reviews are useful to address emergent or unexplored topics.

We conducted a scoping review with a systematic search of health literature during May 2017, in PubMed and PaHO-VHL (Pan American Health Association*-*Virtual Health Library), and two LAC databases, LILACS (Latin American and Caribbean Health Sciences) and SciELO (Scientific Electronic Library Online). We scanned reference lists and citations of the selected papers in Google Scholar to identify additional studies.

For the search strategy, we used combinations of two groups of terms in English and Spanish (only English in PubMed). First, “mining” and “mining industry.” Second, “sexual health,” “reproductive health,” “maternal health,” “HIV,” “sexually transmitted diseases,” “sexually transmitted infections,” “fertility,” and “sexual behavior.” To narrow the search in PubMed and PaHO-VHL, we used the term of exclusion “data mining.”

**Fig. 1:**
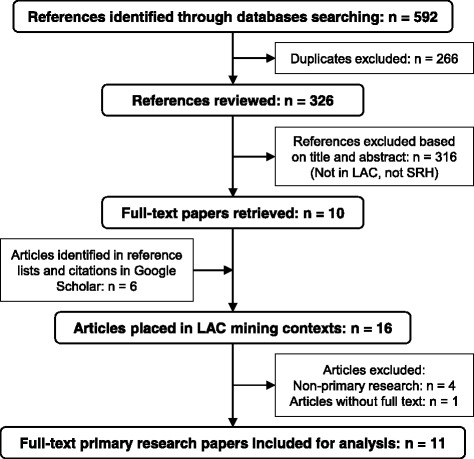
Scoping review selection process

We included only references placed in LAC countries, with no limitation by the date of publication. We excluded articles addressing exclusively environmental and occupational health (e.g., exposure to pollutants, even regarding reproductive health outcomes), health issues different from SRH (e.g., children’s health), and non-health issues. We excluded non-primary research papers (e.g., reports, editorial comments, and letters to the editor). The selection process included the elimination of duplicates, selection of references based on titles and abstracts, identification of full-text papers, selection of additional studies from reference lists and citations in Google Scholar, and final selection of full-text primary research articles (Fig. 1).

Finally, we summarized and discussed the selected articles based on the country and sub-region of the study, key features of the mining operations (minerals, scale, and formality/legality), the purpose of the study, population and sample size, and main findings on SRH.

## Results

The scoping review yielded 592 primary references and retrieved 16 articles placed in LAC countries that focused on SRH (Fig. 1). We eliminated one article without full text and four non-primary papers, which included two recent reviews on health impact assessment related to extractive industries and hydroelectric projects in the region [29, 35]. We finally selected 11 primary research articles for analysis. Table 1 shows the main findings of the scoping review.

**Table 1:** General characteristics and main findings of studies addressing SRH in LAC mining contexts

Authors (year)	Country, sub-region	Mining features	Aims	Population (*n*)	Main SRH findings
Santos et al. (1995) [42]	Brazil, Pará (AR)	Gold ASM/informal	Describe general health conditions in a (mining) community	General population (*n* = 223)	Hep B prevalence = 85%. Syphilis prevalence = 41.6% (males, 33%; females, 9%); the highest among miners (48%) and sex workers (38%).
Souto et al. (1998) [45]	Brazil, Mato Grosso (AR)	Gold^a^	Determine the prevalence and risk factors of Hep B virus among immigrants	General population (*n* = 783)	Hep B prevalence = 54.7%. Significant association between HBV markers and having lived in a gold-mining camp.
Faas et al. (1999) [38]	Venezuela, Bolívar (AR)	Gold Industrial and ASM/informal	Determine the prevalence of HIV/STDs and implement prevention workshops	General population (survey, *n* = 2000; biological tests, *n* = 893)	HIV prevalence = 1%. Syphilis prevalence = 16.6%; highest among female sex workers (29.5%) and health care workers (21.7%). History of STDs = 19.6%.
González and Rodríguez-Acosta (2000) [36]	Venezuela, Bolívar (AR)	Gold^a^	Determine the prevalence of STDs, among other health conditions	Attendants to a local health service (*n* = 166)	Prevalence of syphilis = 6%; gonorrhea = 4.8%; and HIV = 0%. History of STDs = 27.7%.
Souto et al. (2001) [44]	Brazil, Mato Grosso (AR)	Gold^a^	Determine the prevalence and risk factors of Hep B and C (and HBV subtypes)	Population of mine camps (*n* = 520)	Prevalence of Hep B = 82.9% and Hep C = 2.1%. HBsAg positivity was significantly associated with previous STDs.
Palmer et al. (2002) [41]	Guyana (AR)	Gold ASM^a^	Determine the prevalence of HIV	Mineworkers—mostly males (*n* = 216)	HIV prevalence = 6.5%.
Seguy et al. (2008) [43]	Guyana, three regions (AR)	Gold and diamonds^a^	Determine the prevalence of HIV and syphilis	Male mineworkers (*n* = 651)	Prevalence of HIV = 3.9% and syphilis = 6.4%. Knowledge on HIV = 75%, having had casual sex in the last year = 54%, and having had sex with sex workers = 14.8%. HIV was significantly associated with history of syphilis and not having used condom with last casual sex partner.
Miranda et al. (2009) [39]	Brazil, Pará (AR)	Gold ASM^a^	Describe the reproductive profile of and prevalence of STDs among women living in a former mining village	Women attending to a local health facility (*n* = 209)	Prevalence of HIV = 1.9%, gonorrhea = 2.4%, and HPV = 3.8%. History of previous STDs = 11%, having been involved in prostitution = 15.8%, domestic violence reported = 17.7%, having been raped = 10%, and having had an abortion = 29.2%.
Astete et al. (2010) [37]	Peru, Apurimac	Gold, silver, copper, iron Industrial/formal (Pre-exploitation)	Determine the prevalence of infectious diseases, mental health, and environmental pollution in a community surrounding an upcoming mining project	General population (*n* = 453)	No cases of HIV and Hep C or D. Prevalence of syphilis = 1.4% (similar to the national) and Hep B = 7.1% (lower than the national).
Orellana et al. (2013) [40]	Peru, three departments (AR)	Gold ASM/informal	Examine structural factors related to the increased HIV/STD vulnerability among indigenous people	Indigenous population (40 in-depth interviews; nine focus groups, *n* = 98)	Complex interactions between structural factors characterize Amazon rivers in Peru as risk environments for the HIV/STDs spread among indigenous communities.
Castro-Arroyave et al. (2016) [46]	Colombia, Antioquia	Gold Informal^a^	Implement a CBPR intervention of HIV prevention and measure the prevalence of HIV	Community leaders (*n* = 10); general population (*n* = 277; survey and HIV tests, *n* = 183)	No cases of HIV. Prevalence of syphilis = 2.2%. Lack of knowledge, false beliefs, and stigma regarding HIV were identified. Participants increased their knowledge and changed perceptions about HIV issues.

*AR* Amazon Region, *ASM* artisanal and small-scale mining, *CBPR* community-based participatory research, *Hep* hepatitis

^a^Scale and/or formality status not mentioned

### General characteristics of the studies: places, times, and populations

The 11 studies reviewed, conducted between 1995 and 2016 [36–46], were set in gold-mining contexts in South American countries, nine in Amazon areas. One study was set in a period previous to a mining exploitation phase [37] and one in a period of mining decline [39]. The other nine were set in periods of full mining operations. Except for the study by Orellana et al. [40], the investigations used quantitative methods and cross-sectional designs, collecting data through surveys and biological samples for HIV and STDs tests. None of the studies used a longitudinal approach to incorporate temporality in the analyses. However, the study by Astete et al. [37] considered temporality and provided a baseline in a pre-exploitation period to evaluate the health impacts of mining during the subsequent phases. Three studies sampled mostly or exclusively male miners [41, 43, 44], one included only women [39] and one focused on indigenous population [40]. The rest of the studies were sampled from the general population.

### Purposes and main SRH findings of the studies

The 11 studies centered on HIV/STDs as the only SRH concern. The ten quantitative studies focused on measuring the prevalence of HIV and STDs. The study by Castro-Arroyave et al. [46] showed a low prevalence of HIV but a high prevalence of syphilis in a period of mining exploitation; the HIV prevalence contrasts with the high prevalence of HIV found by the rest of the quantitative studies in a similar stage of mining production. Astete et al. [37] described a low prevalence of HIV/STDs in a stage of pre-exploitation. Some studies identified a high prevalence of HIV/STDs among mineworkers [42], sex workers [38, 42], and health care workers [38] than the general population. Three investigations evidenced significant associations between HIV/STDs and individual risk factors such as having lived in a gold-miners camp [45], having history of STDs [43, 44], and not having used a condom with last casual sex partner [43]. Most of the quantitative studies suggested labor migration and mobility as major factors involved in HIV/STD transmission in mining places. Nevertheless, only Souto et al. [45] provided actual evidence on the association between STDs and mobility.

Two studies developed HIV prevention interventions [38, 46]. They used behavioral change approaches aimed to increase knowledge, change attitudes, and reduce stigma about HIV/STDs. Castro-Arroyave et al. [46] found change in perceptions and increases in knowledge among community leaders and other community members who participated in the program.

The study by Orellana et al. [40] provided unique insights into the links between mining and SRH. Qualitative in-depth data enabled the researchers to identify and discuss emerging themes such as population mobility and mixing, sociocultural factors (early sexual initiation, sexual identity, and gender roles), and interpersonal and behavioral factors (forced sex, unprotected sex, and transactional sex among poor women and young indigenous men). Authors concluded that “poverty, cultural beliefs related to health and sexual behaviors, gender inequality, lack of educational and employment opportunities, resource-extraction activities, and population mobility and mixing” interact and shape risk environments for the HIV/STD spread among indigenous communities ([40]: p. 1246).

## Discussion

The literature reviewed about SRH in LAC mining contexts is scarce and provides limited evidence in this area of research in the region. The increased prevalence of HIV/STDs among mineworkers, sex workers, or other population groups that most of the studies showed, along with the reported associations with individual risk factors, match findings from other regions [2, 3]. The low prevalence of HIV/STDs that Castro-Arroyave et al. [46] found in a gold-mining community in Colombia, however, contrasts with the global findings. Two studies provided evidence on the role of labor migration and mobility on the spread of HIV/STDs in LAC mining contexts [40, 45]. Although they are limited, similar findings have been reported globally [2, 9–11].

Based on the findings of this scoping review, we identify four critical gaps in the current knowledge on SRH in LAC mining contexts. First, there is no empirical research that examines SRH broadly beyond the spread of HIV/STDs. No studies have explored SRH issues related to sexual violence against women, adolescents, and children; intimate partner violence; reproductive decision-making; use of contraception; early marriage; teenage pregnancy; abortion; maternal health; or access and use of SRH services. As discussed by Vargas-Riaño et al. in regard to maternal health research in the region [47], the dominant focus on HIV issues seems to respond primarily to research funding interests and not to the health needs of the LAC population. This gap in knowledge is also characteristic of the global research on this matter. Second, there is no empirical research regarding SRH issues in settings different from gold-mining contexts and from Mexico and Central America, the Southern LAC, and the Caribbean. The lack of studies in these other contexts is salient since numerous communities throughout Latin America have experienced the extractive boom [15, 29] with unknown effects on their SRH. Third, there is no empirical research that explores the mechanisms or pathways through which SRH is shaped in LAC mining contexts, particularly in periods of extractive boom. Finally, there is a dearth of investigations about effective SRH interventions in LAC mining contexts. In light of these needs, we discuss two key areas to consider in future research: the mining context and the production of risk in these scenarios.

### The mining context revisited

The study of the relationship between mining and SRH in LAC has overlooked the context where mining takes place. For most of the studies reviewed, the construct *mining* referred either to the setting that delimits the location of research (the *mining* community, the *mining* camp) or an individual sociodemographic variable (the *mineworker* occupation). Astete et al. [37] and Miranda et al. [39] also considered the stage of mining production in their research approach, suggesting differential effects of the time of mining on SRH. Nevertheless, only Orellana et al. [40] explored and reflected on mining and other resource-extraction activities as contextual factors that interact with others to determine a “risk environment” for the spread of HIV/STDs.

The mining context in a period of extractive boom has been commonly characterized by disruptive demographic, environmental, socioeconomic, and gender dynamics triggered by the mining economy. Significant workforce migration to mining places (predominantly male) and high mining wages in mining communities relate to increases in the local demand and prices of land, housing, basic goods, and services, as described in countries like Peru [48]. The increased cost of living and high expectations of short-term returns from the mining economy pull people from rural settings to engage in mining activities and displace traditional livelihoods [49, 50]. Living conditions of migrant workers in camps and mining towns—including single-sex accommodation, isolation, and separation from families—along with large amounts of disposable cash among men have been associated with high demand for alcohol, drugs, and sex services [5, 51]. These factors have in turn been linked to an increase in the number of bars, nightclubs, and brothels [40, 52], as well as rising levels of crime and violence, particularly sexual violence, as observed in gold camps in Surinam [28] and Peru [53].

These dynamics determine a context of unbalanced gendered power relations, where women are disproportionally vulnerable to the environmental, socioeconomic, and health impacts of mining [52]. As reviewed by Jenkins [52], the mining sector often establishes a displacement from subsistence economies towards cash economies controlled by men. Women shift away from their traditional roles to become mineworkers (mainly restricted to processing tasks) or providers of services—including sex. Female mineworkers are paid less than males, even when they are performing heavy tasks like those performed by men [27, 54]. Extreme poverty may lead young girls to get involved in prostitution, bonded labor, and sexual exploitation in places of ASM and informal mining, as described in Amazon countries [27, 28]. Some studies in mining settings worldwide observed that women might engage in transactional sex as a supplementary source of income, basic goods, or favors [4, 8]. Orellana et al. [40] reported transactional sex practices among poor women and indigenous teenage boys in the Peruvian Amazon. In general, women have little access to the economic benefits of mining and become highly dependent on men, resulting in a degradation of their social status and a reinforcement of male privileges [52].

### The production of SRH risk in mining contexts

The simultaneous dynamics often described in mining contexts challenge the way we address the links between mining and SRH in research and intervention. Some studies in South African mining settings showed that prevention interventions typically based on biomedical and behavioral approaches had little impact in reducing or even controlling the spread of HIV [1, 4, 8, 55]. Based on these findings, Desmond et al. [8] argued that conventional approaches—restricted to the individual level of analysis—do not represent adequately the complexity of the social and sexual networks that take place in mining contexts. Instead, Desmond et al. [8] proposed the use of a *high-risk environment* approach which enables structural-level analyses and interventions. The study by Orellana et al. [40] coincides with this perspective as it focused on the structural and contextual production of risk. The authors examined *ecosocial levels* of influence and discussed the *risk environment* as an appropriate category of analysis about the spread of HIV/STDs among indigenous communities. Even though the authors did not explore broad aspects of the SRH—likely occurring in these same risk environments, this approach serves as a point of reference for further exploration about the way SRH is shaped in LAC mining contexts.

### Implications for future research

To address the gaps in knowledge identified, new research efforts must consider key conceptual and methodological challenges. First, they require comprehensive approaches for SRH that include, in addition to the study of HIV and STDs, broader SRH aspects related to sexual violence against women, adolescents and children, intimate partner violence, reproductive decision-making, use of contraception, early marriage, adolescent pregnancy, abortion, maternal health, and access and use of SRH services. Second, future studies must consider the mining context as a critical component of analysis, by examining the demographic, environmental, socioeconomic, and gender dynamics triggered by the mining economy, involved in the production of SRH risk. This also requires taking into account the particularities of the mining operations, i.e., minerals of extraction, stages of mining, scales, and status of formality/legality. Empirical research should consider the use of categories like *risk environment* instead of *high-risk population*, as proposed by Desmond et al. [8] and Orellana et al. [40]; the use of qualitative methods and longitudinal designs for quantitative studies; and the use of multilevel approaches to examine structural, contextual, interpersonal, and individual factors and pathways of influence involved. Finally, future research requires integrating a gender perspective for the examination of the gender roles and gender relations in mining contexts. A gender approach would facilitate the exploration and understanding of phenomena such as the unbalanced gendered power relations as determinants of SRH.

### Limitations

We conducted a scoping literature review looking for peer-reviewed publications. We did not examine gray literature, e.g., dissertations, technical reports, or conference papers. Despite potential drawbacks on availability and quality, gray literature might provide insights on how other researchers and stakeholders (international, government, industry, and civil organizations) have approached the relationship between mining and SRH in LAC. For instance, Organización Internacional del Trabajo, Gobierno de Chile, and SEREMI Tarapacá [56] presented a survey on knowledge, perceptions of risk, attitudes, and practices regarding HIV among mobile mineworkers (*n* = 300), in the copper region of Tarapacá, Chile. Mineworkers showed high levels of knowledge in HIV issues, high demand for commercial sex (during life, 33.1%; during the past year, 13.4%), and little access to condoms (81.4% referred non-availability of condoms in the mining place). These findings, similar to those from Seguy et al. [43], also targeting male mineworkers, provide a background for further research on SRH in mining communities in Chile. At the same time, these results confirm the gaps in knowledge in the LAC region regarding the restricted interest on HIV and individual risk factors associated, as well as the lack of attention to the demographic, environmental, socioeconomic, and gender dynamics linked to the mining economy as key factors determining SRH.

## Conclusions

This scoping review clarifies the current knowledge about SRH in LAC mining contexts and joins the recent reviews by Drewry, Shandro, and Winkler [29] and Pereira et al. [35] towards the understanding and assessment of the health impacts of the extractive industries in the region. We found that available research is scarce and provides limited evidence on SRH in LAC mining contexts. This is significant considering the numerous communities along LAC that have experienced a mining boom during the past decade, with unknown effects on their SRH. The critical gaps identified include little knowledge on (1) broader SRH impacts besides HIV/STDs, (2) SRH in settings different from gold-mining contexts in Amazon countries, (3) mechanisms shaping SRH in LAC mining contexts, and (4) effective interventions in these scenarios. We expect these findings stimulate LAC research teams, stakeholders, and communities interested in sexual and reproductive health and rights, gender equity, environmental justice, occupational health, and community health to advocate for and conduct new investigations on this critical but neglected topic of public health research.
